# Sustained Adoption of an Evidence-Based Treatment: A Survey of Clinicians Certified in Problem-Solving Therapy

**DOI:** 10.1155/2012/986547

**Published:** 2012-09-13

**Authors:** Rebecca M. Crabb, Patricia A. Areán, Mark T. Hegel

**Affiliations:** ^1^Department of Psychiatry, University of California, San Francisco, CA 94143, USA; ^2^Department of Psychiatry, Geisel School of Medicine at Dartmouth, NH 03755, USA

## Abstract

Training models that incorporate case supervision in addition to didactic instruction appear to be effective in maximizing clinicians' proficiency in evidence-based treatments (EBTs). However, it is unknown the extent to which these models promote sustained adoption of EBTs. We describe the results of an online survey on post-training utilization of an EBT, problem-solving therapy (PST), among 40 clinicians highly trained in PST. Seventy-five percent of the survey's 40 respondents reported that they continued to use PST in their clinical practices. Many PST-trained clinicians reported that they had modified the PST protocol in their clinical practices according to patient characteristics or preferences. Considering these results, we recommend emphasizing patient variability and treatment tailoring throughout the training process as a means for promoting clinicians' sustained adoption of EBTs.

## 1. Introduction

Implementing evidence-based treatments (EBTs) into community settings is a well-recognized priority in mental health services research, but the evidence base on implementation strategies that work is still limited [1]. Many factors are theorized to influence the success of implementation efforts, including characteristics of the intervention, inner and outer practice settings, individual clinicians, and the implementation process itself [2]. Training models that incorporate didactic instruction, case supervision, and assessment of fidelity appear to be effective in maximizing clinicians' proficiency in EBTs [3, 4]. However, the extent to which this model of training promotes clinicians' sustained adoption of those interventions is unknown. 

 Problem-solving therapy (PST) is an EBT for major depression [5, 6]. A version of PST was manualized for the Improving Mood-Promoting Access to Collaborative Treatment (IMPACT) study [7], a randomized trial of integrated depression care management for older adults [8]. A rigorous PST training and certification protocol, involving both didactic and case supervision components, was developed to train clinicians for IMPACT and has since been used in other controlled studies [9, 10], as well as community implementation projects.

In the context of ongoing efforts to implement PST and other evidence-based interventions using similar training models, it is important to understand how clinicians apply these treatments in practice. A background in and stronger adherence to the principles and methods of cognitive-behavioral therapy (CBT) has been found to be associated with better patient outcomes in studies of CBT and PST [11, 12]. At the same time, successful community implementation of EBTs is most likely under conditions of “mutual adaptation,” that is, when the treatment can be adapted to the needs of patients, clinicians, and organizations without losing integrity or effectiveness [13]. The aim of this study was thus to “follow” PST-trained clinicians into the community and ask them how frequently they continued to use PST in their clinical practices and in what ways, if any, they had chosen to adapt the PST protocol. 

## 2. Method

### 2.1. Intervention and Training

Problem-solving therapy addresses depression by teaching people a series of steps to approach and manage life problems, thus increasing self-efficacy and improving mood and hopefulness [14]. Using the IMPACT training model, the didactic portion of PST interventionist training consists of the provision of a treatment manual and participation in a day-long workshop with opportunities to role-play PST. The supervised experience component requires trainees to apply the PST approach with 2-3 clients and provide video or audio recordings of the first, third, and sixth sessions with each client to their PST trainer. Trainers review the recordings and provide feedback and case supervision to trainees on each recorded session. Trainers rate trainees on their execution of each step of the PST model using the Problem-Solving Treatment Adherence and Competence scale [15]. Each step is rated on a six point scale as follows: 5 = very good, 4 = good, 3 = satisfactory, 2 = borderline, 1 = poor, and 0 = very poor. Only trainees who demonstrate “satisfactory” performance on their 2-3 cases are certified. Between 2000 and 2009, 66 clinicians from various disciplines have attained certification-level training in PST using the IMPACT training model; 57 of 66 (86%) were trained in PST as part of a research study. 

### 2.2. Participants and Procedure

These 66 clinicians with certification-level training in PST were eligible for the study. Contact information was available for 58 of the 66 eligible clinicians, of whom 40 completed the survey, for a response rate of 69%. We emailed clinicians a description of the study and a link to the survey site, which provided further information and the choice to opt into or out of the survey. Respondents who completed the survey had the option of entering their names into a drawing for a $100 gift certificate to an online bookseller. Clinicians who had not completed the survey 3 weeks after receiving the initial email request were sent a reminder email. Data were collected between May and August 2009. All study procedures were approved by the institutional review board at the University of California, San Francisco (UCSF).

### 2.3. Instrument

We developed the survey with feedback and suggestions from PST trainers at UCSF, Dartmouth, and Duke Universities and pilot tested it with three PST-certified clinicians at UCSF. The survey consisted of a mix of closed- and open-ended questions on clinicians' professional background, training in PST and current use of PST in clinical practice, outside of work on a research study. Frequency distributions are reported for the 8 closed-ended questions (Questions 1–8; see Tables 1 and 2), and qualitative summaries are presented for the 2 open-ended questions (Questions 9–10; described in text). 

## 3. Results

### 3.1. Professional Background and Theoretical Orientation

Table 1 presents details on survey respondents' professional backgrounds and training in PST. Respondents represented a range of professional disciplines and varied considerably in years of postdegree experience, with a range of 2–37 years and a mean of 12.8 years. Nearly half of the respondents who responded to the question on primary theoretical orientation reported that their orientation was cognitive behavioral (18/37; 49%). The others were most likely to identify as psychodynamic (6/37; 16.2%) or eclectic/integrative (8/37; 21.6%). Thirty-five of the 40 respondents (88%) reported having used PST as part of a research study, either currently (18; 45%) or in the past (17; 43%). The amount of time that had elapsed between training and taking part in the survey ranged from several months to 11 years, with a mean of 3.4 years.

### 3.2. Current Use of PST in Clinical Practice

Table 2 presents data on respondents' posttraining use of PST. In response to the close-ended question asking how often clinicians used PST in their clinical practice outside of a research context, thirty of 40 respondents (75%) reported either “frequently” or “occasionally” using PST. All six of the respondents who said that they used PST “not at all” and one of the four who said they used it “rarely” explained, in response to the open-ended question on factors influencing decision to continue using PST in clinical practice, that they do not have a clinical practice outside of a research setting, and therefore did not have the opportunity to use PST. Among the 34 clinicians who still used PST in their clinical practices, over 80% reported that they continued to use each of the seven basic steps of PST. Self-reported adherence was lower with respect to associated elements of the protocol. For example, only 68% reported using a PST homework form. 

All 40 respondents provided comments in response to the open-ended question, “What factors have influenced your decision to continue or not to continue using PST in your clinical practice?” In favor of its continued use, several common themes were noted.

- Clients' needs and preferences (14 comments). For example, “PST can be relevant if they are particularly stuck or wanting a more solution focused intervention for a specific problem.”
- The intervention's focus on concrete change (7 comments). For example, “[PST is] a practical approach that works well for…those less interested in insight or those who are overwhelmed by life circumstances like lack of housing or unemployment.”
- The intervention's seamless ease of integration with other approaches (5 comments), for example, CBT, dialectical behavior therapy (DBT), and motivational interviewing.

Three respondents shared reasons for rarely or never using PST. They included: client disinterest, lack of fit with implementer's theoretical orientation, and poor fit for persons with advanced cognitive impairment (i.e., model too complex).

Thirty six respondents provided comments in response to the second open-ended question, “We know that as clinicians gain experience with PST, they may adapt it to better suit the needs of their clinical population or work setting. What changes have you yourself implemented to the format and process of PST in your clinical practice?” The following themes were noted.

- Adaptations to the PST homework forms (10 comments). For example, respondents described not using the forms at all, using them to explain the concepts but not for homework, or creating new forms to individualize the treatment.
- Integration of PST as one component of an eclectic intervention (7 comments). For example, “I use [PST] in a more ad hoc fashion, without structuring psychotherapy around the PST steps or making the problem solving the primary goal of treatment.”
- Changing the language used to educate clients in the model, for reasons related to mental health stigma (3 comments). For example, “get away from the word “problem” and substitute the words “issues,” “challenges,” or “something that you would like to see changed in your life.”

## 4. Discussion

The current study provided data on the sustained adoption of an evidence-based treatment, problem-solving therapy (PST), among 40 clinicians trained to a high degree of proficiency. Most of the PST-trained clinicians surveyed reported using PST in their clinical practices either frequently or occasionally, and many reported having modified PST according to patient preferences and characteristics. Our findings extend the results of an earlier study, in which nearly all of a group of 11 family medicine residents trained in PST reported they continued to use PST three years after receiving training, often in modified form [15]. 

Limitations of the present study include its small sample size, moderate response rate, and reliance on clinicians' self-reported behavior. Given that almost 90% of clinicians in our study had been trained in PST as part of a research study, it is possible that our sample may have been more highly motivated than clinicians without this background to continue using an evidence-based approach like PST after training. It is also possible that clinicians who responded to the survey were more likely to use PST than those who did not respond. Nevertheless, there was considerable variability within our sample concerning clinicians' theoretical orientation, years of experience, and the manner in which clinicians chose to apply PST in their clinical practices. Indeed, it was interesting that so many clinicians reported modifying PST despite their research background. 

Tailoring an evidence-based treatment according to client needs and preferences, as well as provider expertise and resources, is consistent with the principles of evidence-based behavioral practice. Evidence-based behavioral practice, or EBBP, has been described as a process whereby clinical judgment and client-specific needs are integrated with empirical evidence concerning various treatment approaches in the selection and application of interventions [16]. If, as our results suggest, modification of a structured treatment protocol is common, it may be useful for trainers and supervisors to explicitly discuss how to individualize EBTs for diverse clients while maintaining fidelity to the treatment's core components (cf. the use of a modular approach to training, [17]). Trainers might emphasize the rationale and empirical justification for each component of an EBT so that clinicians can judiciously apply or adapt each component. For example, the empirical evidence supporting the use of “homework assignments” in behavioral therapies [18], including PST [15] might be used to reinforce the rationale for asking clients to work on solving problems outside of sessions. 

Feedback from clinicians with experience using the treatment in the field, such as that provided by the clinicians in our survey, may help to identify treatment components that are easier or more difficult to implement and provide rich examples of creative applications of core components [19]. For example, several PST-trained clinicians noted that they had developed new homework forms to meet the needs of the client and/or setting. In future studies, it would be useful to elicit specific examples of the ways clinicians had adapted PST. The questionnaire could be modified to focus on each of the seven steps of PST in turn, asking about ease and frequency of use as well as examples of modifications they have made to those steps. We might acquire a more objective, in-depth understanding of sustained adoption of PST by observing video or audio tapes of therapy sessions recorded months or years after training. In addition, we might track other factors that may influence implementation, such as characteristics of the clinical population or the setting in which the treatment is provided (cf., [2]).

Our results provide initial support for the IMPACT training model as a means to promote sustained adoption of PST, but must be replicated in larger studies where adherence and modifications to the structured protocol are assessed objectively. Given the documented scarcity of research on what happens to evidence-based treatments after dissemination [4], the current study represents an initial attempt to “follow” an EBT into regular clinical practice. Effective dissemination and implementation of EBTs depend on an understanding of how the therapists who will ultimately deliver those treatments respond to and use the training they receive. 

## Figures and Tables

**Table 1:** Professional background and training in PST.

Characteristic	Survey respondents (*N* = 40 PST-certified clinicians)
Professional background	*n* = 40

Clinical or counseling psychology (PhD, PsyD, or EdD)	18 (45.0%)
Licensed clinical social worker or masters of social work	10 (25.0%)
Masters level nurse or clinical nurse specialist	4 (10.0%)
Other (e.g., MA, MD, MS)	8 (20.0%)
Unknown	0 (0.0%)

Used PST in research	*n* = 40

In the past	18 (45.0%)
Currently	17 (42.5%)
Never	5 (12.5%)

Years clinical experience	*n* = 39

0–5	13 (33.3%)
6–10	9 (23.1%)
11–15	6 (15.4%)
15+	11 (28.2%)

Theoretical orientation	*n* = 37

Cognitive behavioral	18 (48.6%)
Psychodynamic	6 (16.2%)
Eclectic/integrated	8 (21.6%)
Family therapy	1 (2.7%)
Medical	1 (2.7%)
Self psychology	1 (2.7%)
Physical rehabilitation	1 (2.7%)
Early childhood	1 (2.7%)

Years since PST certification	*n* = 40

0–5	22 (55.0%)
6–10	17 (42.5%)
11	1 (2.5%)

Satisfaction with amount of training in PST	*n* = 40

Too much	1 (2.5%)
Too little	1 (2.5%)
Just right	38 (95.0%)

**Table 2:** Survey respondents' current use of PST in clinical practice.

Question	Response options	Number (percent)
Do you still use the following elements of PST in your clinical practice? (*n* = 34 clinicians who still used PST in clinical practice)	Problem orientation	
	Reframe problems as challenges	29 (85.3%)
	Discuss the relationship between depression and problem solving	31 (91.2%)
	Make a problem list at the outset of therapy	26 (76.5%)
	Problem definition	33 (97.1%)
	Goal-setting	32 (94.1%)
	Brainstorming	33 (97.1%)
	Evaluation of solutions	29 (85.3%)
	Action plan	30 (88.2%)
	Evaluation of outcome	30 (88.2%)
	Problem-solving materials	
	Original forms	21 (61.8%)
	Own forms	15 (44.1%)

How often do you use PST (or elements of PST) with clients/patients who you see for therapy OUTSIDE OF a research context? (*n* = all 40 survey respondents)	Frequently	16 (40.0%)
	Occasionally	14 (35.0%)
	Rarely	4 (10.0%)
	Not at all	6 (15.0%)
